# Supplier Selection of Medical Consumption Products with a Probabilistic Linguistic MABAC Method

**DOI:** 10.3390/ijerph16245082

**Published:** 2019-12-12

**Authors:** Guiwu Wei, Cun Wei, Jiang Wu, Hongjun Wang

**Affiliations:** 1 School of Business, Sichuan Normal University, Chengdu 610101, China; 2 School of Statistics, Southwestern University of Finance and Economics, Chengdu 611130, Chinawujiang@swufe.edu.cn (J.W.); 3 School of Economics and Management, Chongqing University of Arts and Sciences, Chongqing 402160, China

**Keywords:** multiple attribute group decision making (MAGDM), probabilistic linguistic term sets (PLTSs), MABAC method, CRITIC method, combined weights, medical consumption products, supplier selection

## Abstract

In order to obtain an optimal medical consumption product supplier, the integration of combined weights and multi-attributive border approximation area comparison (MABAC) under probabilistic linguistic sets (PLTSs) has offered a novel integrated model in which the CRiteria Importance Through Intercriteria Correlation (CRITIC) method is employed for calculating the objective weights of various attributes and the MABAC method with PLTSs is used to acquire the final ranking result of a medical consumption product supplier. Additionally, so as to indicate the applicability of the devised method, this model is confirmed by a numerical case for the supplier selection of medical consumption products. Some comparative studies are made with some existing methods. The proposed method can also successfully select suitable alternatives in other selection problems.

## 1. Introduction

Along with ever growing complication and ambiguity of decision making issues and the fuzziness of human subjective cognition, it is increasingly arduous for DMs (decision makers) to offer exact judgments. Thus, to obtain an optimal choice of a qualitative MADM (Multiple attribute decision making) that can be utilized to easily depict the qualitative assessment of information, Herrera and Martinez [1] devised linguistic term sets (LTSs) for calculating with words. Herrera and Martinez [2] utilized linguistic two-tuples algorithms to address multi-granular linguistic information. Herrera-Viedma et al. [3] presented a novel information retrieval systems (IRSs) model under the two-tuple linguistic environment. Li et al. [4] proposed and utilized two novel two-tuple linguistic models that were distribution function models. Estrella et al. [5] proposed Flintstones, which tackled linguistic MADM issues within a two-tuple linguistic environment; they also designed the Flintstones website, which kept a helpful numerical example and dataset repository for diverse linguistic decision making issues. In recent years, more and more studies have combined the two-tuple linguistic model with interval numbers [6,7], intuitionistic fuzzy sets (IFSs) [8,9], Pythagorean fuzzy sets (PFSs), hesitant fuzzy sets (HFSs) [10,11,12,13], and bipolar fuzzy sets (BFSs). Above all, Rodriguez et al. [14] put introduced hesitant fuzzy linguistic term sets (HFLTSs) on the basis of HFSs [15] and LTSs [16]. The HFLTS method is an extremely powerful tool for expressing DMs’ linguistic assessment in a more elastic way. This tool permits DMs to employ some possible LTSs to estimate a linguistic variable. Wei et al. [17] worked out some operations of HFLTSs and possibility degree mathematical formulas for HFLTSs. Wu et al. [18] defined compromised solutions for multiple attribute group decision making (MAGDM) by using HFLTSs. Gou et al. [19] gave entropy measures for MADM under the HFLTSs, ultimately getting over the hesitant fuzzy linguistic generalized dice similarity measures to tackle MADM issues. Wang et al. [20] proposed likelihood-based TODIM algorithms with multi-hesitant linguistic information to assess logistics outsourcing based on classical TODIM algorithms [21,22,23]. Liao et al. [24] provided the VIKOR (VIseKriterijumska Optimizacija I KOmpromisno Resenje) method [25,26,27,28] for qualitative MADM under HFLTSs. Zhang et al. [29] developed a new process of reaching a consensus in MAGDM with HFLTSs. 

However, current HFLTSs fail to take the significant of weight information of each possible linguistic term into consideration, and then all the possible linguistic terms are treated under the assumption that their weights are equivalent. Clearly, this is not a practical case. Though DMs could hesitate among several possible linguistic terms, they may have some preferences in certain situations, so these linguistic terms could be treated by using different weight information. In consideration of this reality, Pang et al. [30] came up with the probabilistic linguistic term sets (PLTSs) to conquer these limits, and they defined a framework for ranking PLTSs by score and deviation. Gou and Xu [31] put forward operational laws for HFLEs (hesitant fuzzy linguistic elements) and PLTSs with two equivalent shifting mathematical functions. Cheng et al. [32] investigated the group decision-making of venture capitalists with interplay within a probabilistic linguistic environment. Xie et al. [33] researched an incomplete hybrid PLTS. Lin et al. [34] developed the ELECTRE (ELimination Et Choix Traduisant la REalité) II method to handle PLTSs for edge computing. Liao et al. [35] also researched the novel operations of PLTSs and devised an ELECTRE III method with PLTSs. Feng et al. [36] constructed a probabilistic linguistic QUALIFLEX method that can provide a comparison of possibility degree. Chen et al. [37] used MULTIMOORA (Multi-Objective Optimization on the basis of Ratio Analysis plus the full multiplicative form) for a cloud-based ERP (Enterprise Resource Planning) system selection with PLTSs. Kobina et al. [38] defined some power operators with PLTSs to tackle the MAGDM with classical power aggregation operators [39,40]. Liang et al. [41] designed a probabilistic linguistic grey relational analysis (PL-GRA) for the MAGDM based on the geometric Bonferroni mean [42,43]. Liao et al. [44] came up with a linear programming approach to tackle the MADM with PLTSs. Zhang et al. [45] proposed a consensus algorithm to analyze the GDM with probabilistic linguistic preference relations. Zhai et al. [46] developed probabilistic multi-granular linguistic vector-term sets. Song and Hu [47] established preference relation with PLTSs in complex environments. Song and Li [48] proposed the the consensus process, in which MGPFLPRs (multi-granular probabilistic fuzzy linguistic preference relations) are utilized to express the preference information of sub-groups. To settle these issues, Song and Li [49] proposed an LGDM method in which incomplete information is more relevant for multi-stakeholders to stand for their evaluation; three normalizing algorithms were presented to obtain the entire PLTSs based on three kinds of risk attitudes: optimistic, pessimistic and neutral. Lu et al. [50] designed the TOPSIS (Technique for Order of Preference by Similarity to Ideal Solution) method for the probabilistic linguistic MAGDM with entropy weight for the supplier selection of new agricultural machinery products.

The MABAC (multi-attributive border approximation area comparison) approach was first put forward by Pamucar and Cirovic [51], and it derives distance measures between every possible alternative and bored approximation area (BAA). Pamucar et al. [52] combined the interval-rough AHP (Analytical Hierarchy Process) with the MABAC methods to assess university website construction. Pamucar et al. [53] modified the MABAC methods with uncertain fuzzy-rough numbers. Sharma et al. [54] investigated the multiple criteria evaluation framework by using the rough AHP-MABAC method. Xue et al. [55] studied the MABAC model to select material under interval-valued IFSs. Peng and Dai [56] designed algorithms for an interval neutrosophic MADM on the basis of some methods, including the MABAC, the similarity measure, and EDAS (Evaluation based on Distance from Average Solution). Peng and Yang [57] devised the Pythagorean fuzzy MABAC approach in the MAGDM. Sun et al. [58] tackled the projection-based MABAC model under HFLTSs for patients’ prioritization. 

From our perspective, studies of the MABAC method have failed to explore MAGDM problems under PLTSs. Thus, the employing MABAC method in the MAGDM is an attractive research topic that can rank and acquire optimal alternatives under PLTSs. Meanwhile, while noting taking different weights into account influences the sorting results, a novel method is proposed to decide the weights through integrating subjective elements with objective ones. To obtain such goals, the major research contribution can be described as follows: (1) The modified MABAC is extended by PLTSs. (2) The probabilistic linguistic MABAC (PL-MABAC) method is developed to tackle MAGDM problems with PLTSs. (3) Taking different weights into account influences the sorting results, a combined weight method is devised by integrating subjective weights with objective weights that can be separated in two aspects: The first one is the subjective weight and the other is the objective weight, which can be derived by the CRiteria Importance Through Intercriteria Correlation (CRITIC) method. These subjective weight calculating methods emphasize the DMs’ preference information, while the objective information is neglected. The objective weight calculating methods fail to consider DMs’ preference information. In other words, the DMs’ risk attitudes are ignored. The obvious highlight of this integrated weight includes both subjective and objective weights. As a consequence, an integrated method is put forward to derive attribute weights. (4) A simple case for the supplier selection of medical consumption products is proposed to prove the developed approach. (5) Some comparative studies are given with the PLWA (probabilistic linguistic weighted average) operator, the PL-TOPSIS method, and the PL-GRA method to prove the legitimacy of the PL-MABAC method. In the meantime, the modified PL-GRA method is devised to simultaneously emphasize the shape similarity degree from the PIS (positive ideal solution) and NIS (negative ideal solution).

In order to reach these research goals, the other sections are arranged as follows. Section 2 presents some fundamental concepts connected with PLTSs. In Section 3, the MABAC method is introduced for MAGDM problems under PLTSs. In Section 4, a numerical example for the supplier selection of medical consumption products is shown, and some comparative studies are devised. The study ends with some conclusions in Section 5.

## 2. Preliminaries

The concept of PLTS [30] is indicated as below.

**Definition 1** **[59].** *Let*$L=\left\{l_{\alpha }|\alpha =-\theta ,\cdots ,-2,-1,0,1,2,\cdots \theta \right\}$*be an LTS, and the linguistic terms*$l_{\alpha }$*can convey equivalent information to*β*, which is represented with transformation function*ϕ:

(1) $$\varphi :\left[l_{-\theta },l_{\theta }\right]\to \left[0,1\right],\varphi \left(l_{\alpha }\right)=\frac{\alpha +\theta }{2\theta }=\beta$$

β*can also be expressed the equivalent one to the linguistic terms,*$l_{\alpha }$*, which is denoted with the shifting function*$g^{-1}$:

(2) $$\varphi^{-1}:\left[0,1\right]\to \left[l_{-\theta },l_{\theta }\right],\;\varphi^{-1}\left(\beta \right)=l_{\left(2\beta -1\right)\theta }=l_{\alpha }$$

**Definition 2** **[30].** *Given an LTS*$L=\left\{l_{j}|j=-\theta ,\cdots ,-2,-1,0,1,2,\cdots \theta \right\}$*, a PLTS is devised as:*

(3) $$L\left(p\right)=\left\{l^{\left(\phi \right)}\left(p^{\left(\phi \right)}\right)|l^{\left(\phi \right)}\in L,p^{\left(\phi \right)}\geq 0,\phi =1,2,\cdots ,\#L\left(p\right),\sum_{\phi =1}^{\#L\left(p\right)}p^{\left(\phi \right)}\leq 1\right\}$$

*where*$l^{\left(\phi \right)}\left(p^{\left(\phi \right)}\right)$*is*ϕth*linguistic term*$l^{\left(\phi \right)}$*linked with corresponding probability value*$p^{\left(\phi \right)}$*, and*#L(p)*is the corresponding length of linguistic terms in*L(p)*. The linguistic terms*$l^{\left(\phi \right)}$ in L(p) *are listed in ascending order.*

*In order to easy computation, Pang, Wang and Xu [30] normalized the PLTS*L(p)*as*$\tilde{L}\left(p\right)=\left\{l^{\left(\phi \right)}\left({\tilde{p}}^{\left(\phi \right)}\right)|l^{\left(\phi \right)}\in L,{\tilde{p}}^{\left(\phi \right)}\geq 0,\phi =1,2,\cdots ,\#L\left(\tilde{p}\right),\sum_{\phi =1}^{\#L\left(p\right)}{\tilde{p}}^{\left(\phi \right)}=1\right\}$*, where*${\tilde{p}}^{\left(\phi \right)}=p^{\left(\phi \right)}/\sum_{\phi =1}^{\#L\left(p\right)}p^{\left(\phi \right)}$*for all*$\phi =1,2,\cdots ,\#L\left(\tilde{p}\right)$.

**Definition 3** **[30].** *Let* $L=\left\{l_{\alpha }|\alpha =-\theta ,\cdots ,-1,0,1,\cdots \theta \right\}$ *be an LTS and* ${\tilde{L}}_{1}\left(\tilde{p}\right)=\left\{l_{1}{}^{\left(\phi \right)}\left({\tilde{p}}_{1}{}^{\left(\phi \right)}\right)|\phi =1,2,\cdots ,\#{\tilde{L}}_{1}\left(\tilde{p}\right)\right\}$ *and* ${\tilde{L}}_{2}\left(\tilde{p}\right)=\left\{l_{2}{}^{\left(\phi \right)}\left({\tilde{p}}_{2}{}^{\left(\phi \right)}\right)|\phi =1,2,\cdots ,\#{\tilde{L}}_{2}\left(\tilde{p}\right)\right\}$ *be two PLTSs, where* $\#{\tilde{L}}_{1}\left(\tilde{p}\right)$ *and* $\#{\tilde{L}}_{2}\left(\tilde{p}\right)$ *are the corresponding numbers of PLTS* $\#{\tilde{L}}_{1}\left(\tilde{p}\right)$ *and* $\#{\tilde{L}}_{2}\left(\tilde{p}\right)$ *, respectively. If* $\#{\tilde{L}}_{1}\left(\tilde{p}\right)>\#{\tilde{L}}_{2}\left(\tilde{p}\right)$ *, then add* $\#{\tilde{L}}_{1}\left(\tilde{p}\right)-\#{\tilde{L}}_{2}\left(\tilde{p}\right)$ *linguistic terms to* ${\tilde{L}}_{2}\left(\tilde{p}\right)$ *. Furthermore, the newly added linguistic terms should be the smallest linguistic term in* ${\tilde{L}}_{2}\left(\tilde{p}\right)$ *, and the probabilities of newly added linguistic terms should be zero.*

**Definition 4** **[30].** *For a PLTS*$\tilde{L}\left(\tilde{p}\right)=\left\{l_{}{}^{\left(\phi \right)}\left({\tilde{p}}_{}{}^{\left(\phi \right)}\right)|\phi =1,2,\cdots ,\#{\tilde{L}}_{}\left(\tilde{p}\right)\right\}$*, the score*$s\left(\tilde{L}\left(\tilde{p}\right)\right)$*and deviation*$\sigma \left(\tilde{L}\left(\tilde{p}\right)\right)$ of $\tilde{L}\left(\tilde{p}\right)$ *is devised as:*

(4) $$s\left(\tilde{L}\left(\tilde{p}\right)\right)=\sum_{\phi =1}^{\#{\tilde{L}}_{}\left(\tilde{p}\right)}\;\varphi \left(\tilde{L}\left(\tilde{p}\right)\right){\tilde{p}}_{}{}^{\left(\phi \right)}/\sum_{\phi =1}^{\#{\tilde{L}}_{}\left(\tilde{p}\right)}{\tilde{p}}_{}{}^{\left(\phi \right)}$$

(5) $$\sigma \left(\tilde{L}\left(\tilde{p}\right)\right)=\sqrt{\sum_{\phi =1}^{\#{\tilde{L}}_{}\left(\tilde{p}\right)}{\left(\;\varphi \left(\tilde{L}\left(\tilde{p}\right)\right){\tilde{p}}_{}{}^{\left(\phi \right)}-s\left(\tilde{L}\left(\tilde{p}\right)\right)\right)}^{2}}/\sum_{\phi =1}^{\#{\tilde{L}}_{}\left(\tilde{p}\right)}{\tilde{p}}_{}{}^{\left(\phi \right)}$$

*By using the Equations (4) and (5), the order relation between two PLTSs is devised as follows. (1) If*$s\left({\tilde{L}}_{1}\left(\tilde{p}\right)\right)>s\left({\tilde{L}}_{2}\left(\tilde{p}\right)\right)$*, then*${\tilde{L}}_{1}\left(\tilde{p}\right)>{\tilde{L}}_{2}\left(\tilde{p}\right)$*; (2) if*$s\left({\tilde{L}}_{1}\left(\tilde{p}\right)\right)=s\left({\tilde{L}}_{2}\left(\tilde{p}\right)\right)$*, then*$\sigma \left({\tilde{L}}_{1}\left(\tilde{p}\right)\right)=\sigma \left({\tilde{L}}_{2}\left(\tilde{p}\right)\right)$*and*${\tilde{L}}_{1}\left(\tilde{p}\right)={\tilde{L}}_{2}\left(\tilde{p}\right)$*; if*$\sigma \left({\tilde{L}}_{1}\left(\tilde{p}\right)\right)<\sigma \left({\tilde{L}}_{2}\left(\tilde{p}\right)\right)$*, then*${\tilde{L}}_{1}\left(\tilde{p}\right)>{\tilde{L}}_{2}\left(\tilde{p}\right)$.

**Definition 5** **[34].** *Let*$L=\left\{l_{\alpha }|\alpha =-\theta ,\cdots ,-1,0,1,\cdots \theta \right\}$*be an LTS. Let*${\tilde{L}}_{1}\left(\tilde{p}\right)=\left\{l_{1}{}^{\left(\phi \right)}\left({\tilde{p}}_{1}{}^{\left(\phi \right)}\right)|\phi =1,2,\cdots ,\#{\tilde{L}}_{1}\left(\tilde{p}\right)\right\}$*and*${\tilde{L}}_{2}\left(\tilde{p}\right)=\left\{l_{2}{}^{\left(\phi \right)}\left({\tilde{p}}_{2}{}^{\left(\phi \right)}\right)|\phi =1,2,\cdots ,\#{\tilde{L}}_{2}\left(\tilde{p}\right)\right\}$*be two PLTSs with*$\#{\tilde{L}}_{1}\left(\tilde{p}\right)=\#{\tilde{L}}_{2}\left(\tilde{p}\right)=\#\tilde{L}\left(\tilde{p}\right)$*, so the Hamming distance*$d\left({\tilde{L}}_{1}\left(\tilde{p}\right),{\tilde{L}}_{2}\left(\tilde{p}\right)\right)$*between*${\tilde{L}}_{1}\left(\tilde{p}\right)$ and ${\tilde{L}}_{2}\left(\tilde{p}\right)$ *is devised as:*

(6) $$d\left({\tilde{L}}_{1}\left(\tilde{p}\right),{\tilde{L}}_{2}\left(\tilde{p}\right)\right)=\frac{\sum_{\phi =1}^{\#{\tilde{L}}_{1}\left(\tilde{p}\right)}\left|{\tilde{p}}_{1}{}^{\left(\phi \right)}\;\varphi \left(l_{1}{}^{\left(\phi \right)}\right)-{\tilde{p}}_{2}{}^{\left(\phi \right)}\;\varphi \left(l_{2}{}^{\left(\phi \right)}\right)\right|}{\#\tilde{L}\left(\tilde{p}\right)}$$

## 3. MABAC Method for Probabilistic Linguistic MAGDM Problems 

In such section, we propose a novel probabilistic linguistic MABAC (PL-MABAC) method for MAGDM problems. The following mathematical notations were utilized to solve the probabilistic linguistic MAGDM issues. Assume that there is a collection of alternatives $AL=\left\{AL_{1},AL_{2},\cdots ,AL_{m}\right\}$ and $AT=\left\{AT_{1},AT_{2},\cdots ,AT_{n}\right\}$ with a weight vector $w=\left(w_{1},w_{2},\cdots ,w_{n}\right)$, where $w_{j}\in \left[0,1\right]$,$\sum_{j=1}^{n}w_{j}=1$ and q exerts $E=\left\{E_{1},E_{2},\cdots ,E_{q}\right\}$. Suppose that there are n qualitative attribute $AT=\left\{AT_{1},AT_{2},\cdots ,AT_{n}\right\}$ and their values are assessed by each expert and depicted as linguistic expressions $l_{ij}^{k}\left(i=1,2,\cdots ,m,j=1,2,\cdots ,n,k=1,2,\cdots ,q\right)$. 

Then, the PL-MABAC approach is designed to tackle MAGDM problems. The concrete calculating procedure is involved in the following steps:

**Step 1.** Shift cost attributes into beneficial attributes. If $L=\left\{l_{\alpha }|\alpha =-\theta ,\cdots ,-1,0,1,\cdots \theta \right\}$ is an LTS, then the cost attribute value is $l_{\alpha }$ and the corresponding beneficial attribute value is $l_{-\alpha }$.

**Step 2.** Shift linguistic assessing values $l_{ij}^{k}\left(i=1,2,\cdots ,m,j=1,2,\cdots ,n,k=1,2,\cdots ,q\right)$ into PLTSs $l_{ij}^{}{}^{\left(\phi \right)}\left(p_{ij}^{}{}^{\left(\phi \right)}\right),\phi =1,2,\cdots ,\#L_{ij}^{}\left(p\right)$ and build the assessing matrix $L={\left(L_{ij}^{}\left(p\right)\right)}_{m\times n}$ with PLTSs, $L_{ij}^{}\left(p\right)=\left\{l_{ij}^{}{}^{\left(\phi \right)}\left(p_{ij}^{}{}^{\left(\phi \right)}\right)|\phi =1,2,\cdots ,\#L_{ij}^{}\left(p\right)\right\}$(i=1,2,⋯,m,j=1,2,⋯,n).

**Step 3.** Derive the normalized assessing matrix $\tilde{L}={\left({\tilde{L}}_{ij}^{}\left(\tilde{p}\right)\right)}_{m\times n}$ with PLTSs, ${\tilde{L}}_{ij}^{}\left(\tilde{p}\right)=\left\{l_{ij}^{}{}^{\left(\phi \right)}\left({\tilde{p}}_{ij}^{}{}^{\left(\phi \right)}\right)|\phi =1,2,\cdots ,\#L_{ij}^{}\left(\tilde{p}\right)\right\}$(i=1,2,⋯,m,j=1,2,⋯,n).

**Step 4.** Compute the combined weight information for attributes.

An essential method called CRiteria Importance Through Intercriteria Correlation (CRITIC) was initially devised by [60], and it is introduced to decide the objective weights of attributes that take the correlations between attributes into consideration. Then, a novel method is proposed to decide the attribute weights by integrating subjective weights with objective weights; these weights can be separated in two aspects: The first aspect is the subjective weights’ methods, and the other is the objective weights’ methods that can be derived with the CRITIC method. Subsequently, the detailed computing procedures of this combined weight method are given as follows.

1. Build the correlation coefficient matrix $CCM={\left(cc_{jt}\right)}_{n\times n}$ by computing the correlation coefficient between attributes.
  (7) $$\begin{array}{c} cc_{jt}=\frac{\sum_{i=1}^{m}\left(\left(\sum_{\phi =1}^{\#{\tilde{L}}_{1}\left(\tilde{p}\right)}{\tilde{p}}_{ij}{}^{\left(\phi \right)}\;\varphi \left(l_{ij}{}^{\left(\phi \right)}\right)-p_{j}^{\left(\phi \right)}\varphi \left(l_{j}^{\left(\phi \right)}\right)\right)\right)\left(\left(\sum_{\phi =1}^{\#{\tilde{L}}_{1}\left(\tilde{p}\right)}{\tilde{p}}_{it}{}^{\left(\phi \right)}\;\varphi \left(l_{it}{}^{\left(\phi \right)}\right)-p_{t}^{\left(\phi \right)}\varphi \left(l_{t}^{\left(\phi \right)}\right)\right)\right)}{\sqrt{\sum_{i=1}^{m}{\left(\sum_{\phi =1}^{\#{\tilde{L}}_{1}\left(\tilde{p}\right)}{\tilde{p}}_{ij}{}^{\left(\phi \right)}\;\varphi \left(l_{ij}{}^{\left(\phi \right)}\right)-p_{j}^{\left(\phi \right)}\varphi \left(l_{j}^{\left(\phi \right)}\right)\right)}^{2}}\sqrt{\sum_{i=1}^{m}{\left(\sum_{\phi =1}^{\#{\tilde{L}}_{1}\left(\tilde{p}\right)}{\tilde{p}}_{it}{}^{\left(\phi \right)}\;\varphi \left(l_{it}{}^{\left(\phi \right)}\right)-p_{t}^{\left(\phi \right)}\varphi \left(l_{t}^{\left(\phi \right)}\right)\right)}^{2}}},\\ j,t=1,2,\ldots ,n \end{array}$$
  where $l_{j}^{}{}^{\left(\phi \right)}\left(p_{j}^{}{}^{\left(\phi \right)}\right)=\frac{\sum_{i=1}^{m}l_{ij}^{\left(\phi \right)}}{m}\left(\frac{\sum_{i=1}^{m}p_{ij}^{\left(\phi \right)}}{m}\right)$ and $l_{t}^{}{}^{\left(\phi \right)}\left(p_{t}^{}{}^{\left(\phi \right)}\right)=\frac{\sum_{i=1}^{m}l_{it}^{\left(\phi \right)}}{m}\left(\frac{\sum_{i=1}^{m}p_{it}^{\left(\phi \right)}}{m}\right)$.
2. Derive the standard deviation of attribute.
  (8) $$sd_{j}=\sqrt{\frac{1}{m}\sum_{i=1}^{m}{\left(\sum_{\phi =1}^{\#{\tilde{L}}_{1}\left(\tilde{p}\right)}{\tilde{p}}_{ij}{}^{\left(\phi \right)}\varphi \left(l_{ij}{}^{\left(\phi \right)}\right)-p_{j}^{\left(\phi \right)}\varphi \left(l_{j}^{\left(\phi \right)}\right)\right)}^{2}},j=1,2,\ldots ,n,$$
3. Compute the objective weights.
  (9) $$ow_{j}=\frac{sd_{j}\sum_{t=1}^{n}\left(1-cc_{jt}\right)}{\sum_{j=1}^{n}\left(sd_{j}\sum_{t=1}^{n}\left(1-cc_{jt}\right)\right)},j=1,2\ldots ,n,$$
  where $ow_{j}\in \left[0,1\right]$ and $\sum_{j=1}^{n}ow_{j}=1$.
4. Determine the combined weights. Suppose that the subjective weight directly given by the DMs is $sw=\left(sw_{1},sw_{2},\cdots ,sw_{n}\right)$, where $sw_{j}\in \left[0,1\right]$, j=1,2,⋯,n, $\sum_{j=1}^{n}sw_{j}=1$. The objective weight is calculated by Equation (9) directly, is $ow=\left(ow_{1},ow_{2},\cdots ,ow_{n}\right)$, where $ow_{j}\in \left[0,1\right]$, j=1,2,⋯,n, $\sum_{j=1}^{n}ow_{j}=1$. Therefore, the combined weights of attributes $cw=\left(cw_{1},cw_{2},\cdots ,cw_{n}\right)$ could be defined:
  (10) $$cw_{j}=\frac{ow_{j}\times sw_{j}}{\sum_{j=1}^{n}ow_{j}\times sw_{j}}$$
  where $cw_{j}\in \left[0,1\right]$, j=1,2,⋯,n, $\sum_{j=1}^{n}cw_{j}=1$.

The objective and subjective weight values are merged by a nonlinear weighted comprehensive approach. In accordance with the multiplier impact, the higher the values of the subjective and objective weight are, the higher the combined weight are, or vice versa. In the meantime, we may derive that Equation (10) overcomes the drawback of taking either subjective or objective impact elements into account. The distinct merit of Equation (10) is that both objective and subjective weights can be reflected by the attribute weights and alternatives ranks.

**Step 5.** Solve the probabilistic linguistic border approximation area (PLBAA) matrix $\mathrm{PLBAA}={\left(PLBAA_{j}\right)}_{1\times n}$. The PLBAA for all attributes is derived according to Equations (11)–(13).

(11) $$\mathrm{PLBAA}={\left(PLBAA_{j}\right)}_{1\times n}$$

(12) $$PLBAA_{j}=\left\{{\bar{l}}_{j}^{\left(\phi \right)}\left({\bar{p}}_{j}^{\left(\phi \right)}\right)|\phi =1,2,\cdots ,\#{\tilde{L}}_{ij}^{}\left(\tilde{p}\right)\right\}$$

(13) $${\bar{l}}_{j}^{\left(\phi \right)}\left({\bar{p}}_{j}^{\left(\phi \right)}\right)=\varphi^{-1}\left(\sqrt[{m}]{\prod_{i=1}^{m}\varphi \left(l_{ij}{}^{\left(\phi \right)}\right)}\right)\left(\sqrt[{m}]{\prod_{i=1}^{m}p_{ij}^{\left(\phi \right)}}\right)$$

**Step 6.** Compute the probabilistic linguistic Hamming distance matrix $PLHD={\left(PLHD_{ij}\right)}_{m\times n}$ from PLBAA by using Equations (14) and (15).

(14) $$PLHD_{ij}=\{\begin{array}{ll} d\left({\tilde{L}}_{ij}^{}\left(\tilde{p}\right),PLBAA_{j}\right)w_{j}, & if\;\left(\sum_{\phi =1}^{\#L_{ij}\left(\tilde{p}\right)}\varphi \left(l_{ij}^{\left(\phi \right)}\right)\left(p_{ij}^{\left(\phi \right)}\right)-\varphi \left({\bar{l}}_{j}^{\left(\phi \right)}\right)\left({\bar{p}}_{j}^{\left(\phi \right)}\right)\right)/\#{\tilde{L}}_{ij}\left(\tilde{p}\right)>0,\;\\ 0, & if\;\left(\sum_{\phi =1}^{\#L_{ij}\left(\tilde{p}\right)}\varphi \left(l_{ij}^{\left(\phi \right)}\right)\left(p_{ij}^{\left(\phi \right)}\right)-\varphi \left({\bar{l}}_{j}^{\left(\phi \right)}\right)\left({\bar{p}}_{j}^{\left(\phi \right)}\right)\right)/\#{\tilde{L}}_{ij}\left(\tilde{p}\right)=0,\\ -d\left({\tilde{L}}_{ij}^{}\left(\tilde{p}\right),PLBAA_{j}\right)w_{j}, & if\;\left(\sum_{\phi =1}^{\#L_{ij}\left(\tilde{p}\right)}\varphi \left(l_{ij}^{\left(\phi \right)}\right)\left(p_{ij}^{\left(\phi \right)}\right)-\varphi \left({\bar{l}}_{j}^{\left(\phi \right)}\right)\left({\bar{p}}_{j}^{\left(\phi \right)}\right)\right)/\#{\tilde{L}}_{ij}\left(\tilde{p}\right)<0. \end{array}$$

(15) $$d\left({\tilde{L}}_{ij}^{}\left(\tilde{p}\right),LBAA_{j}\right)=\left(\sum_{\phi =1}^{\#L_{ij}\left(\tilde{p}\right)}\left|\varphi \left(l_{ij}^{\left(\phi \right)}\right)\left(p_{ij}^{\left(\phi \right)}\right)-\varphi \left({\bar{l}}_{j}^{\left(\phi \right)}\right)\left({\bar{p}}_{j}^{\left(\phi \right)}\right)\right|\right)/\#{\tilde{L}}_{ij}\left(\tilde{p}\right)$$

where the distance measure d is defined as Equation (6).

Now, if $PLHD_{ij}=0$ then the alternative $AL_{i}$ belongs to the border approximation area PLBAA. If $PLHD_{ij}>0$, it belongs to the probabilistic linguistic upper approximation area (PLUAA). If $PLHD_{ij}<0$, it belongs to the probabilistic linguistic lower approximation area (PLUAA). Clearly, the PLUAA includes the best alternative $AL^{+}$, and, on the contrary, the PLUAA includes the anti-ideal alternative $AL^{-}.$

**Step 7.** Compute the probabilistic linguistic score value (PLSV) of the alternatives.

(16) $${\mathrm{PLSV}}_{i}=\sum_{j=1}^{n}PLHD_{ij},\;i=1,2,\ldots ,m,$$

**Step 8.** Sort the alternatives according to ${\mathrm{PLSV}}_{i}\left(i=1,2,\ldots ,m\right)$; the higher value of ${\mathrm{PLSV}}_{i}\left(i=1,2,\ldots ,m\right)$, the better the alternative is. 

## 4. A Case Analysis and Comparative Analysis

### 4.1. A Case Analysis

The supplier terms of an enterprise are undoubtedly very important and will be an even more important influence on the quality of a vendor’s business in the future. Thus, these terms will affect the business of purchasing, production, inventory and sales, and so on. Additionally, the relationship between suppliers and future enterprises is not a simple relationship between management and managed—suppliers will become strategic partner companies, making it a win–win relationship. Thus, the preliminary evaluation and selection of a supplier is quite important. Medical supply products have their own characteristics to distinguish them from other types of products that can be distinguished from their production, transportation, marketing, and other aspects. Therefore, medical supply product supplier requirements, such as the degree of environmental cleaner and production level, are different. If there is FDA (Food and Drug Administration) approval, the company-established supplier evaluation system is selected according to the characteristics of the product itself with medical supplies, a standard can be more adapted to the company’s development and enhance the company’s core competitiveness [61,62,63]. The relationship between traditional companies and suppliers are not simply trading relations, because corporate decision-makers think that suppliers cannot change, though you can change the home cooperative attitude of suppliers who are not very friendly in a buyer’s market. when With the progress of economic and social development, a simple buyer–seller relationship cannot meet the needs of enterprise survival and development because some vendors already have their own technology and core competencies. A buyer’s market gradually turns into a seller’s market, so this time we need a new type of partnership—strategic partnerships with suppliers are mutually beneficial and are common developments [64,65,66,67]. Corporate supplier evaluation and the management of late selections is a gradual process that needs to be optimized in order to gradually phase out the bad, to allow the good to join in, and to form a dynamic balance so as to enable enterprises to maintain long-term competitiveness. It can be seen that the supplier selection of medical consumption products is a classical MADM or MAGDM issue [68,69,70,71,72,73,74]. Consequently, a case study for the supplier selection of medical consumption products is presented to illustrate the proposed method. There are five potential medical consumption product suppliers $AL_{i}\left(i=1,2,3,4,5\right)$ to choose. There are four attributes selected by these experts to evaluate the five potential medical consumption product suppliers: (1) AT_1_ is the environmental improvement quality; (2) AT_2_ is the transportation cost of suppliers; (3) AT_3_ is the green image; and (4) AT_4_ is the environmental competencies. The transportation cost (AT_2_) is not a beneficial attribute, but others are beneficial attributes. These five potential medical consumption product suppliers $AL_{i}\left(i=1,2,3,4,5\right)$ must be assessed by using the LTSs

$$\begin{array}{l} L=\{l_{-3}=extremely\;poor(EP),l_{-2}=very\;poor(VP),\\ \;\;\;\;\;\;\;l_{-1}=poor(P),l_{0}=medium(M),\;l_{1}=good(G),\\ \;\;\;\;\;\;\;l_{2}=very\;good(VG),l_{3}=extremely\;good(EG)\} \end{array}$$

by the five DMs within the above four beneficial attributes, as listed in Table 1, Table 2, Table 3, Table 4 and Table 5.

In the following, we make use of the PL-MABAC method developed for medical consumption product supplier selection. 

**Step 1.** Shift the cost attribute AT_2_ into a beneficial attribute. If the cost attribute value is $\;l_{\tau }$, then the corresponding beneficial attribute value is $\;l_{-\tau }\left(\tau =-3,-2,-1,0,1,2,3\right)$ (See Table 6, Table 7, Table 8, Table 9 and Table 10).

**Step 2.** Shift the linguistic variables into an assessing matrix with PLTSs (Table 11).

**Step 3.** Figure out the normalized assessing matrix with PLTSs (Table 12).

**Step 4.** Compute the combined weight values for the attributes. Firstly, as could be seen by using Equation (9), the objective weights of attributes were: $ow_{1}=0.2269,\;ow_{2}=0.2827,\;ow_{3}=0.2375$ and $ow_{4}=0.2529$. From here, suppose that the subjective weights of the attributes were: $sw_{1}=0.3,\;sw_{2}=0.2,\;sw_{3}=0.4$ and $sw_{4}=0.1$. Finally, through Equation (10), the combined weight values for attributes were found to be: $cw_{1}=0.2779,\;cw_{2}=0.2309,\;cw_{3}=0.3879$ and $cw_{4}=0.1033$.

**Step 5.** Solve the PLBAA = ${\left(PLBAA_{j}\right)}_{1\times 4}$ for all attributes according to Equations (11)–(13) (Table 13).

**Step 6.** Compute the PLHD (probabilistic linguistic Hamming distance) from PLBAA by using Equations (14) and (15), as displayed in Table 14.

**Step 7.** Compute the PLSV_i_ (i = 1,2,3,4,5) with Equation (16). The calculation results are shown in Table 15.

**Step 8.** According to PLSV_i_ (i = 1,2,3,4,5), we could sort all the medical consumption product suppliers. Obviously, the order could be found to be: AL_4_ > AL_3_ > AL_2_ > AL_5_ > AL_1_, the optimal medical consumption product supplier is AL_4_, and the bad medical consumption product supplier is AL_1_.

### 4.2. Comparative Analysis

Then, we made a comparison of our proposed approach with PLWA operator [30], the PL-TOPSIS method [30] and the PL-GRA method [41] (let ρ=0.5). The detailed computing results are shown in Table 16.

Based on the above studies and analysis, it can be observed that these four methods derived the same optimal medical consumption product supplier AL_4_ and the same bad medical consumption product supplier AL_1_. In the meantime, the sorting results in the proposed method are in complete agreement with the sorting result in the two methods in reference [30]. The PL-MABAC method we proposed in this paper was confirmed to be logical and valid. These four methods have their own advantages: (1) The PL-TOPSIS method simultaneously emphasizes the distance closeness degree from the PIS and NIS; (2) the PL-GRA method emphasizes the shape similarity degree from the PIS, but it does not emphasize the shape similarity degree from the NIS, thus making the sorting results in [41] slightly different from the other three methods; (3) the PLWA operator emphasizes the group influences degrees; (4) our proposed PL-MABAC method is an efficient and reliable decision making tool with direct computation algorithms and a steady solution.

For the PL-GRA approach proposed by Liang, Kobina and Quan [41], we modified the PL-GRA method, which simultaneously emphasizes the shape similarity degree from the PIS and NIS; the corresponding computing results are listed in Table 17. From the computing results in Table 17, it can be seen that the sorting results in [41] were slightly in complete agreement with the sorting result in the above four methods.

## 5. Conclusions

In this paper, we broadened the MABAC approach to the MAGDM with PLTSs. Firstly, the basic definition, comparative formula, and Hamming distance of PLTSs were briefly reviewed. Then, the modified MABAC method was explained to address probabilistic linguistic MAGDM problems with combined weight information; this method’s crucial trait is that it only highlights the distance closeness degree from the border approximation area for all attributes. In the end, a practical case study about medical consumption product supplier selection was employed to prove the modified method, and some comparative analyses were also designed to verify the method’s applicability in real-world MAGDM problems. The method proposed in tis paper is a very efficient tool for exploiting novel decision-making software and novel decision support systems with PLTSs, and it can solve diverse problems. At the same time, the proposed method can successfully contribute to the selection of suitable alternatives in other selection issues. In this research, the MABAC method, which is also an efficient MADM or MAGDM method, was designed to tackle uncertain decision-making issues. Someday, the proposed models and algorithms with PLTSs could be applied in the other uncertain decision making scenarios [75,76,77,78,79] and many other unpredictable and ambiguous environments [80,81,82,83,84].

## Figures and Tables

**Table 1:** Linguistic assessing matrix by the first DM (decision maker).

Alternatives	AT_1_	AT_2_	AT_3_	AT_4_
AL_1_	EG	VP	VP	VP
AL_2_	VP	VP	G	VG
AL_3_	EG	VP	P	EG
AL_4_	VG	G	EG	G
AL_5_	EG	EP	P	P

**Table 2:** Linguistic assessing matrix by the second DM.

Alternatives	AT_1_	AT_2_	AT_3_	AT_4_
AL_1_	G	EP	VP	EP
AL_2_	VP	VP	G	EG
AL_3_	VG	P	P	EG
AL_4_	VG	VG	VG	EG
AL_5_	G	EP	P	P

**Table 3:** Linguistic assessing matrix by the third DM.

Alternatives	AT_1_	AT_2_	AT_3_	AT_4_
AL_1_	G	EP	VP	P
AL_2_	P	VP	G	G
AL_3_	EG	P	M	EG
AL_4_	VG	VG	EG	VG
AL_5_	G	EP	P	VP

**Table 4:** Linguistic assessing matrix by the fourth DM.

Alternatives	AT_1_	AT_2_	AT_3_	AT_4_
AL_1_	EG	VP	VP	VP
AL_2_	VP	VP	EG	EG
AL_3_	EG	P	P	EG
AL_4_	VG	VG	VG	VG
AL_5_	G	EP	P	VP

**Table 5:** Linguistic assessing matrix by the fifth DM.

Alternatives	AT_1_	AT_2_	AT_3_	AT_4_
AL_1_	EG	EP	VP	EP
AL_2_	P	VP	EG	VG
AL_3_	EG	P	P	EG
AL_4_	VG	G	EG	VG
AL_5_	G	EP	P	VP

**Table 6:** Linguistic assessing matrix by the first DM.

Alternatives	AT_1_	AT_2_	AT_3_	AT_4_
AL_1_	EG	VG	VP	VP
AL_2_	VP	VG	G	VG
AL_3_	EG	VG	P	EG
AL_4_	VG	P	EG	G
AL_5_	EG	EG	P	P

**Table 7:** Linguistic assessing matrix by the second DM.

Alternatives	AT_1_	AT_2_	AT_3_	AT_4_
AL_1_	G	EG	VP	EP
AL_2_	VP	VG	G	EG
AL_3_	VG	G	P	EG
AL_4_	VG	VP	VG	EG
AL_5_	G	EG	P	P

**Table 8:** Linguistic assessing matrix by the third DM.

Alternatives	AT_1_	AT_2_	AT_3_	AT_4_
AL_1_	G	EG	VP	P
AL_2_	P	VG	G	G
AL_3_	EG	G	M	EG
AL_4_	VG	VP	EG	VG
AL_5_	G	EG	P	VP

**Table 9:** Linguistic assessing matrix by the fourth DM.

Alternatives	AT_1_	AT_2_	AT_3_	AT_4_
AL_1_	EG	VG	VP	VP
AL_2_	VP	VG	EG	EG
AL_3_	EG	G	P	EG
AL_4_	VG	VP	VG	VG
AL_5_	G	EG	P	VP

**Table 10:** Linguistic assessing matrix by the fifth DM.

Alternatives	AT_1_	AT_2_	AT_3_	AT_4_
AL_1_	EG	EG	VP	EP
AL_2_	P	VG	EG	VG
AL_3_	EG	G	P	EG
AL_4_	VG	P	EG	VG
AL_5_	G	EG	P	VP

**Table 11:** Assessing matrix with probabilistic linguistic sets (PLTSs).

**Alternatives**	**AT_1_**	**AT_2_**
AL_1_	$\left\{l_{1}\left(0.4\right),\;l_{3}\left(0.6\right)\right\}$	$\left\{l_{-3}\left(0.6\right),\;l_{-2}\left(0.4\right)\right\}$
AL_2_	$\left\{l_{-2}\left(0.6\right),\;l_{-1}\left(0.4\right)\right\}$	$\left\{l_{-2}\left(1\right)\right\}$
AL_3_	$\left\{l_{2}\left(0.2\right),\;l_{3}\left(0.8\right)\right\}$	$\left\{l_{-2}\left(0.2\right),\;l_{-1}\left(0.8\right)\right\}$
AL_4_	$\left\{l_{2}\left(1\right)\right\}$	$\left\{l_{1}\left(0.4\right),\;l_{2}\left(0.6\right)\right\}$
AL_5_	$\left\{l_{1}\left(0.8\right),\;l_{3}\left(0.2\right)\right\}$	$\left\{l_{-3}\left(1\right)\right\}$
**Alternatives**	**AT_3_**	**AT_4_**
AL_1_	$\left\{l_{-2}\left(1\right)\right\}$	$\left\{l_{-3}\left(0.4\right),\;l_{-2}\left(0.4\right),\;l_{-1}\left(0.2\right)\right\}$
AL_2_	$\left\{l_{1}\left(0.6\right),\;l_{3}\left(0.4\right)\right\}$	$\left\{l_{1}\left(0.2\right),\;l_{2}\left(0.4\right),\;l_{3}\left(0.4\right)\right\}$
AL_3_	$\left\{l_{-1}\left(0.8\right),\;l_{0}\left(0.2\right)\right\}$	$\left\{l_{3}\left(1\right)\right\}$
AL_4_	$\left\{l_{2}\left(0.4\right),\;l_{3}\left(0.6\right)\right\}$	$\left\{l_{1}\left(0.2\right),\;l_{2}\left(0.6\right),\;l_{3}\left(0.2\right)\right\}$
AL_5_	$\left\{l_{-1}\left(1\right)\right\}$	$\left\{l_{-2}\left(0.6\right),\;l_{-1}\left(0.4\right)\right\}$

**Table 12:** Normalized assessing matrix with PLTSs.

**Alternatives**	**AT_1_**	**AT_2_**
AL_1_	$\left\{l_{1}\left(0\right),l_{1}\left(0.4\right),l_{3}\left(0.6\right)\right\}$	$\left\{l_{2}\left(0\right),l_{2}\left(0.4\right),l_{3}\left(0.6\right)\right\}$
AL_2_	$\left\{l_{-2}\left(0\right),l_{-2}\left(0.6\right),l_{-1}\left(0.4\right)\right\}$	$\left\{l_{2}\left(0\right),l_{2}\left(0\right),l_{2}\left(1\right)\right\}$
AL_3_	$\left\{l_{2}\left(0\right),l_{2}\left(0.2\right),l_{3}\left(0.8\right)\right\}$	$\left\{l_{1}\left(0\right),l_{1}\left(0.8\right),l_{2}\left(0.2\right)\right\}$
AL_4_	$\left\{l_{2}\left(0\right),l_{2}\left(0\right),l_{2}\left(1\right)\right\}$	$\left\{l_{-2}\left(0\right),l_{-2}\left(0.6\right),l_{-1}\left(0.4\right)\right\}$
AL_5_	$\left\{l_{1}\left(0\right),l_{1}\left(0.8\right),l_{3}\left(0.2\right)\right\}$	$\left\{l_{3}\left(0\right),l_{3}\left(0\right),l_{3}\left(1\right)\right\}$
**Alternatives**	**AT_3_**	**AT_4_**
AL_1_	$\left\{l_{-2}\left(0\right),l_{-2}\left(0\right),l_{-2}\left(1\right)\right\}$	$\left\{l_{-3}\left(0.4\right),l_{-2}\left(0.4\right),l_{-1}\left(0.2\right)\right\}$
AL_2_	$\left\{l_{1}\left(0\right),l_{1}\left(0.6\right),l_{3}\left(0.4\right)\right\}$	$\left\{l_{1}\left(0.2\right),l_{2}\left(0.4\right),l_{3}\left(0.4\right)\right\}$
AL_3_	$\left\{l_{-1}\left(0\right),l_{-1}\left(0.8\right),l_{0}\left(0.2\right)\right\}$	$\left\{l_{3}\left(0\right),l_{3}\left(0\right),l_{3}\left(1\right)\right\}$
AL_4_	$\left\{l_{2}\left(0\right),l_{2}\left(0.4\right),l_{3}\left(0.6\right)\right\}$	$\left\{l_{1}\left(0.2\right),l_{2}\left(0.6\right),l_{3}\left(0.2\right)\right\}$
AL_5_	$\left\{l_{-1}\left(0\right),l_{-1}\left(0\right),l_{-1}\left(1\right)\right\}$	$\left\{l_{-2}\left(0\right),l_{-2}\left(0.6\right),l_{-1}\left(0.4\right)\right\}$

**Table 13:** Probabilistic linguistic border approximation area (PLBAA) for all attributes.

	PLBAA
AT_1_	$\left\{l_{0.3145}\left(0.0000\right),l_{0.3145}\left(0.0000\right),l_{1.6440}\left(0.5210\right)\right\}$
AT_2_	$\left\{l_{0.5944}\left(0.0000\right),l_{0.5944}\left(0.0000\right),l_{1.4777}\left(0.5448\right)\right\}$
AT_3_	$\left\{l_{-0.5978}\left(0.0000\right),l_{-0.5978}\left(0.0000\right),l_{-0.0698}\left(0.5448\right)\right\}$
AT_4_	$\left\{l_{-3.0000}\left(0.0000\right),l_{-0.2759}\left(0.0000\right),l_{0.8664}\left(0.3641\right)\right\}$

**Table 14:** PLHD matrix.

Alternatives	AT_1_	AT_2_	AT_3_	AT_4_
AL_1_	0.0429	0.0405	−0.0129	−0.0035
AL_2_	−0.0157	0.0328	0.0690	0.0218
AL_3_	0.0522	0.0226	0.0130	0.0263
AL_4_	0.0398	−0.0133	0.0863	0.0206
AL_5_	0.0306	0.0457	0.0087	0.0000

**Table 15:** Probabilistic linguistic score value (PLSV) for all alternatives.

Alternatives	AL_1_	AL_2_	AL_3_	AL_4_	AL_5_
PLSV	0.0671	0.1079	0.1141	0.1334	0.0849

**Table 16:** Order by using diverse methods.

Methods	Order	Optimal Alternative	Bad Alternative
PLWA operator [30]	AL_4_ > AL_3_ > AL_2_ > AL_5_ > AL_1_	AL_4_	AL_1_
PL-TOPSIS method [30]	AL_4_ > AL_3_ > AL_2_ > AL_5_ > AL_1_	AL_4_	AL_1_
PL-GRA method [41]	AL_4_ > AL_3_ > AL_2_ > AL_5_ > AL_1_	AL_4_	AL_1_
PL-MABAC method	AL_4_ > AL_3_ > AL_2_ > AL_5_ > AL_1_	AL_4_	AL_1_

**Table 17:** The revised probabilistic linguistic grey relational analysis (PL-GRA) method.

Methods	Order
Grey relational coefficient from the PIS	$\varepsilon_{1}^{+}=0.5239,\varepsilon_{2}^{+}=0.6055,\varepsilon_{3}^{+}=0.6502,\varepsilon_{4}^{+}=0.7316,\varepsilon_{5}^{+}=0.5913$
Grey relational coefficient from the NIS	$\varepsilon_{1}^{-}=0.7348,\varepsilon_{2}^{-}=0.6172,\varepsilon_{3}^{-}=0.5128,\varepsilon_{4}^{-}=0.5727,\varepsilon_{5}^{-}=0.5987$
Relative relational degree from the PIS	$\varepsilon_{1}^{}=0.4162,\varepsilon_{2}^{}=0.4952,\varepsilon_{3}^{}=0.5591,\varepsilon_{4}^{}=0.5548,\varepsilon_{5}^{}=0.4969$
Ordering	AL_3_ > AL_4_ > AL_5_ > AL_2_ > AL_1_
